# Efficacy of snap-needle patch therapy in pediatric epilepsy: a case study

**DOI:** 10.3389/fnhum.2025.1618266

**Published:** 2025-10-10

**Authors:** Jia Shi, Yu Gong, Yuanyuan Deng, Zhen Ma, ZiChun Xiao, HanYing Tan, WeiLan Qin

**Affiliations:** ^1^The Second Clinical Hospital of Beijing, University of Chinese Medicine, Beijing, China; ^2^School of Acupuncture and Moxibustion, Beijing University of Chinese Medicine, Beijing, China

**Keywords:** benign epilepsy of childhood with central temporal spikes (BECTS), traditional Chinese medicine (TCM), snap-needle, acupuncture, case report

## Abstract

**Backgrounds:**

Epilepsy is a prevalent neurological disorder in early childhood, often characterized by genetic predisposition and diverse clinical manifestations. Benign epilepsy of childhood with central temporal spikes (BECTS) is the most common form of self-limited focal epilepsy (SeLFE) syndrome in children, accounting for approximately 6%–7% of all childhood epilepsy cases (Wirrell et al., 2011; Camfield et al., 1996). We report a case of a child who was treated with additional snap-needle for 3 months on top of poorly controlled oral medication alone, who stopped having seizures and gradually reduced the dose of oral medication and finally stopped, and was followed up for 1 year after stopping the medication without any recurrence.

**Case summary:**

A 10-years-old male had his first generalized tonic-clonic seizure (GTCS) at age 7 during nocturnal sleep. Following this event, the parents sought medical evaluation, and an electroencephalogram (EEG) showed abnormal wave patterns. Over the following 3 years, the child had 2–3 GTCS annually; despite multiple adjustments to antiepileptic drug (AED) therapy, GTCS frequency indicated disease progression. However, after starting snap-needle patch therapy [guided by Traditional Chinese Medicine (TCM) principles], the child remained seizure-free. Continuous monitoring has been conducted to date. We present this case of a pediatric patient with epilepsy: oral medication failed to control GTCS, but snap-needle patch therapy achieved successful seizure management, after which oral medication was discontinued.

**Conclusion:**

After a systematic literature search, this study represents the first case report of BECTS treated with snap-needle therapy (needle size: 0.220 mm × 1.5 mm). In this case, the child’s GTCSs resolved following the addition of snap-needle therapy, and oral antiepileptic medication was gradually reduced to complete discontinuation; no seizure recurrence was observed within 1 year of follow-up. It is important to acknowledge that the contributions of medication adjustments and the self-limiting nature of BECTS cannot be ruled out in this case. However, based on the observations here, we propose the hypothesis that snap-needle intervention is likely associated with seizure remission. Although the mechanism underlying snap-needle therapy for BECTS remains unclear, the child achieved a QOLCE-16-C score of 98.4 at the end of treatment; during the 1-year follow-up, the number of nighttime sleep disruptions also decreased from 2 to 5 episodes per year to 0. These outcomes surpass those typically achievable through pharmacological intervention alone.

## 1 Introduction

Epilepsy is a prevalent neurological disorder characterized by an abnormal imbalance between excitatory and inhibitory activities within neuronal networks (Fisher et al., 2005). Self-limited focal epilepsy (SeLFE) syndrome constitutes approximately 25% of all pediatric epilepsy cases (Panayiotopoulos et al., 2008). Benign epilepsy of childhood with central temporal spikes (BECTS) represents the most prevalent form of SeLFE, constituting approximately 6%–7% of all pediatric epilepsy cases (Wirrell et al., 2011; Camfield et al., 1996). BECTS typically manifests in early school-aged children (Loiseau and Beaussart, 1973), and may have a hereditary component (Pal et al., 2016; Vears et al., 2012).

Current therapeutic strategies for epilepsy primarily involve pharmacological agents that modulate ion channels or neurotransmitter systems to achieve clinical efficacy (Rogawski et al., 2016). Sodium channel blockers, such as levetiracetam, phenobarbital, and phenytoin sodium, are considered first-line treatments (Rogawski et al., 2016). In cases of persistent status epilepticus unresponsive to oral medications, intravenous administration of benzodiazepines or anesthetics may be warranted (Loiseau and Beaussart, 1973). Nonetheless, certain medications, including barbiturates, may pose long-term detrimental effects on the developing brain (Borowicz-Reutt et al., 2023). Despite the administration of oral or intravenous medications, 37% of patients do not experience relief (Brodie and Dichter, 1996). Patients who exhibit an inadequate response to initial antiepileptic drug (AED) treatment are at a heightened risk of developing refractory epilepsy (Kwan and Brodie, 2000). Childhood and adolescence represent critical periods for bone development, and individuals undergoing long-term AED therapy (or initiating such therapy), demonstrate increased rates of bone loss (Pack, 2008). The developing brains of children are particularly susceptible to the sedative and anticholinergic neurological effects of antiepileptic drugs; When used as part of a multidrug combination therapy, these effects may exacerbate cognitive decline (Jankovic and Dostic, 2012) thereby impairing normal neurodevelopment in pediatric populations.

Amidst ongoing efforts to advance medical treatments, snap-needle therapy, a novel acupuncture technique rooted in traditional Chinese medicine (TCM), is gaining attention in modern clinical practice. Unlike traditional acupuncture, snap-needle therapy involves the superficial insertion of needles at acupuncture points, which are retained for extended durations. This approach minimizes disruption to daily activities while providing continuous acupoint stimulation. For epilepsy management, snap needles for epilepsy are utilized to stimulate acupuncture points, thereby exerting a distal therapeutic effect. Specifically, the stimulation of acupuncture points via snap needles activates sensory neurons, which subsequently interact with the central nervous system (Magnusson et al., 1994). This interaction triggers physiological pathways that regulate both the brain and peripheral systems. Derived from The Yellow Emperor’s Classic of Internal Medicine this microneedling technique is increasingly recognized for its unique therapeutic potential within the context of modern neuroscience.

A systematic literature search confirmed this is the first case report of BECTS treated with snap-needle therapy (needle size: 0.220 mm × 1.5 mm); no similar studies with this therapy and indication were identified.

## 2 Case presentation

### 2.1 Case presentation

A 10-years-old male patient was referred to our hospital by his parents due to the onset of generalized tonic-clonic seizure (GTCS) at the age of 7, occurring with a frequency of 2–3 episodes annually. The patient’s family history reveals potential genetic predispositions. He has a half-sister who experienced two febrile convulsions at the age of 5, which did not recur, and she is currently developing normally. The patient’s father experienced one GTCS during childhood, which was neither clearly diagnosed nor treated. The patient was born at 34 weeks of gestation via uncomplicated vaginal delivery, with a birth weight of 3,000 g. His developmental milestones were achieved within normal limits, including head control at 4 months, independent walking and single-word speech at 1 year, and short sentence formation at 2 years; his (developmental quotient, DQ) was 88 at 5 years.

At the age of 7, the patient began experiencing generalized tonic-clonic seizures (GTCSs) characterized by the following symptoms: approximately 30 min into nocturnal sleep, the patient exhibited sudden limb extension and tension, deviation of the mouth corners to the left, salivary frothing, and upward squinting of the eyes toward the left. This was followed by bilateral upper limb convulsions and an unresponsiveness to family members’ prompts, with the episode lasting over 10 min. Postictal symptoms included mild breath-holding, which gradually subsided, and the patient’s mental status returned to baseline. The initial GTCS occurred without a preexisting fever. Over the subsequent 6 days, the patient experienced 2 additional GTCSs of a similar nature. These episodes were closely associated with sleep-wake transition period.

The patient and his parents presented to the Department of Neurology at a pediatric hospital, where the patient underwent electroencephalogram (EEG) monitoring and brain magnetic resonance imaging (MRI). The MRI results did not reveal any significant abnormalities. However, based on the EEG findings and clinical manifestations, the child was diagnosed with BECTS and initiated on levetiracetam tablets for epilepsy management. The prescribed dosage was 0.375 g orally in the morning and 0.5 g orally in the evening.

Between the ages of 8 and 9, the patient experienced 3 and 2 GTCSs, respectively. These episodes occurred approximately 15 min after falling asleep and were characterized by upward-leftward eye deviation, mild salivation with mouth opening, and subsequent generalized limb convulsions lasting ∼3 min. Postictal recovery was uneventful, with the patient returning to a normal state of consciousness and reporting no discomfort. During this period, the patient’s EEG was conducted at a pediatric hospital, leading to an adjustment in the levetiracetam dosage to 0.75 g in the morning and 1 g in the evening.

At the age of 10, the patient experienced two consecutive generalized tonic-clonic seizures (GTCSs) on the 2 days preceding the consultation. These episodes were characterized by upward-leftward eye deviation, mouth opening, and mild salivation approximately 10 min after falling asleep. This was followed by marked limb convulsions and a temporary unresponsiveness to familial prompts. Each episode lasted ∼ 10 min and resolved spontaneously, and was followed by the patient’s return to baseline mental status.

### 2.2 Clinical examination

On physical examination, the 10-years-old male patient had a height of 141 cm, weight of 51 kg, and body mass index (BMI) of 25.7 kg/m^2^. His motor development was generally normal, speech was intact, and intellectual development was within normal limits, although he ranked in the lower-middle of his class academically.

### 2.3 Electroencephalogram

**FIGURE 1:**
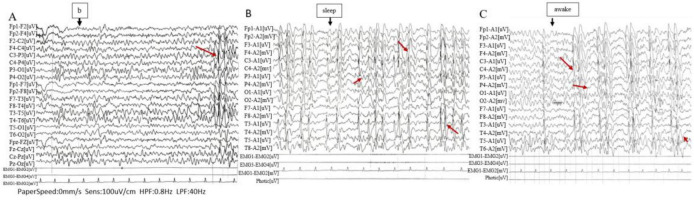
Electroencephalogram (EEG). **(A)** High-amplitude sharp and slow waves are observed in the central-temporal region (Rolandic waves). **(B,C)** Medium to high amplitude sharp waves, spike waves, spike-slow waves, and polyspike waves are frequently detected in the primary leads, predominantly in the right central, parietal, and temporal regions, with potential propagation to other leads (closely related to the sleep-wake transition).

Prior to treatment, the male patient, was admitted to a pediatric hospital where he underwent an EEG examination. The EEG findings indicated abnormal waveforms characterized by asynchrony and frequent occurrences of spikes, polyspikes, sharp waves, and diffuse waves. These anomalies were predominantly observed in the bilateral central and parietal regions, as well as the middle and posterior temporal leads. They had the potential to propagate to adjacent leads during all stages of wakefulness and sleep. Notably, the pathological waveforms were more pronounced during the sleep phase. The EEG background activity was considered abnormal, with complete suppression of alpha waves when the patient was awake, regardless of whether his eyes were open or closed. Pathological waves were also detected during hyperventilation, although flash stimulation was not performed. During sleep, symmetrical bilateral wave crests and hammer waves were observed. The right central, parietal, and temporal regions exhibited medium to high amplitude spiking, spiking slow waves, and polyspiking waves, which could spread to other leads, with the central temporal lead being the most affected (Figure 1).

### 2.4 Diagnosis, treatment, and follow-up

**FIGURE 2:**
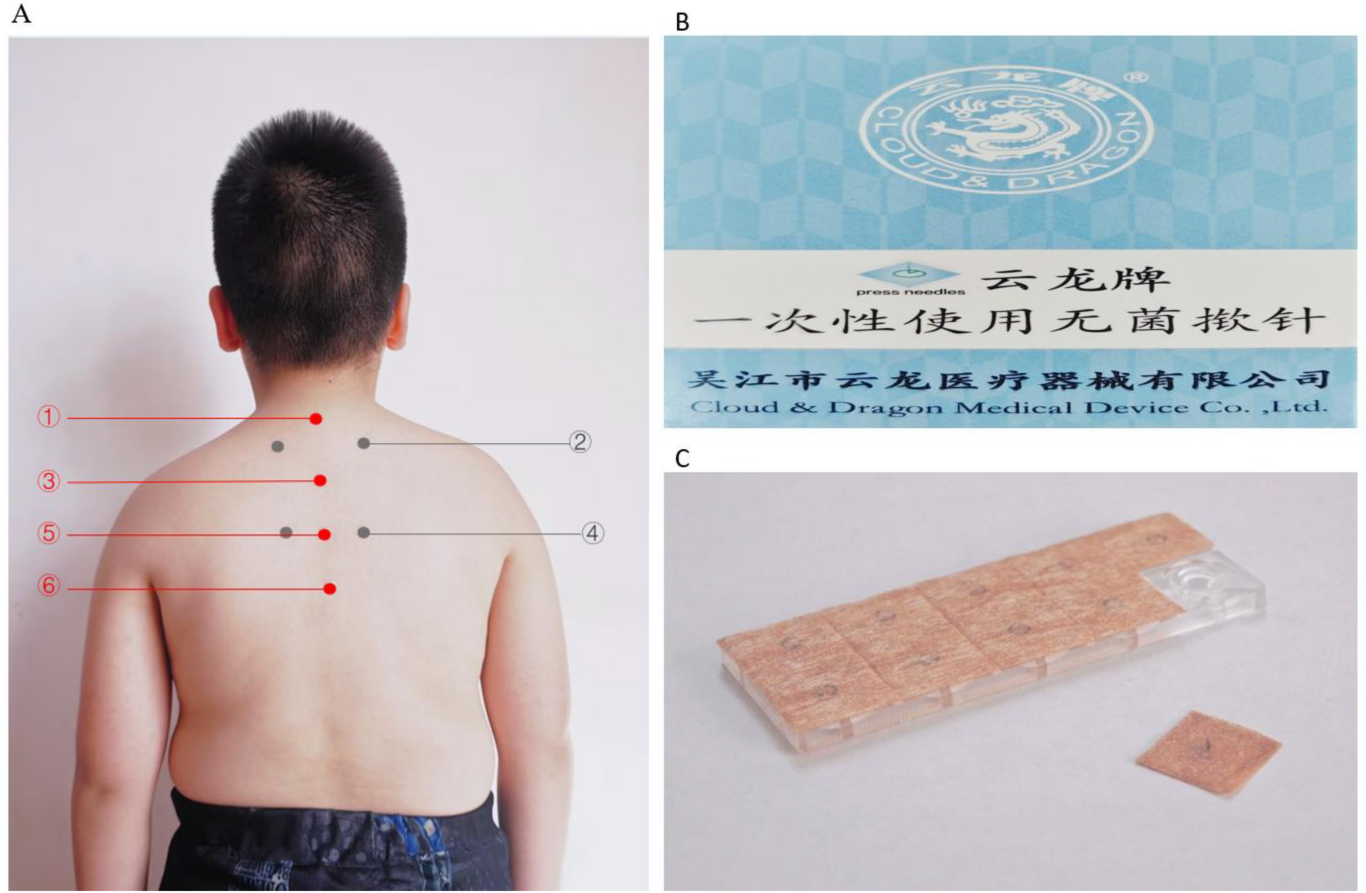
**(A)** The specified positions and the pressing needles utilized. (Apply ➀ + ➂ + ➄ + ➅ + ➁ / ➃ (bilaterally) at each point, where ➁ and ➃ are applied alternately each time. **(B)** Packaging labeled in Chinese and English, reading “Cloud & Dragon Medical Device Co., Ltd.” with “press needles” indicated. **(C)** Close-up of a transparent plastic case containing acupuncture press needles, with one needle sheet partially removed.

Considering the patient’s history, symptoms, and EEG findings, an acupuncturist with 19 years of clinical experience prescribed a 3-month course of snap-needle treatment. The selected acupuncture points included the DU14, BL13, DU11, BL18, DU8, DU6 (Figure 2 and Table 1). Snap-needle stimulation facilitates the propagation of electrical signals generated at these points along the afferent nerve fibers, which then travel through the dorsal roots into the spinal cord sensory signals are transmitted to the central nervous system via the spinothalamic tracts and the dorsal column-medial lemniscal pathway. The thalamus functions acts as a relay station, facilitating the processing and integration of these signals into various regions, including the somatosensory cortex, insula, cingulate cortex, and limbic system, thereby triggering neurotransmitter release and modulating neural activity (Zhang et al., 2014). In the snap-needle treatment procedure, a single-use sterile snap-needle is selected (Figure 2). Initially, the local skin on the patient’s back is disinfected. The snap-needle is then aligned with the designated acupoint and pressed for 30 s at per acupoint. The snap-needle remains in place for 24 h, during which the patient’s parents are instructed to apply pressure to the points three times daily (at 09:00, 16:00, and 20:00) for 30 s each time. The patient is instructed to avoid exposing the snap-needle site to liquids and to refrain from water contact for 4 h after snap-needle removal minimizing the risk of infection. The acupoint locations are illustrated in the accompanying figure. Snap-needle therapy is administered weekly; the same acupoints or areas are not always reused during a 3-months period. Treatment points are adjusted promptly based on the patient’s condition. The specific selection of acupoints is shown in Figure 2 and Table 1.

**TABLE 1:** Location/channel distribution/procedure.

Serial number	Cranial and periocular acupoints	Location	Channel distribution	Operation
➀	Dazhui (DU14)	On the back, in the depression below the spinous process of the 7th cervical vertebra on the posterior midline	The Du Meridian	➀ Disinfect the local skin on the patient’s back.
➁	Feishu (BL13)	On the back, 1.5 cun lateral to the posterior midline, below the spinous process of the 3rd thoracic vertebra.	Bladder Meridian (of foot – Taiyang)	➁ Align the tip of the intradermal needle with the corresponding point.
➂	Shendao (DU11)	On the back, in the depression below the spinous process of the 5th thoracic vertebra on the posterior midline.	The Du Meridian	➂ Press on each acupoint for 30 s after attaching the needle.
➃	Ganshu (BL18)	On the back, 1.5 cun lateral to the posterior mid - line, below the spinous process of the 9th thoracic vertebra.	Bladder Meridian (of foot – Taiyang)	➃ Remove the needle after it has been retained for 1 to 2 days.
➄	Jinsuo (DU8)	On the back, in the depression below the spinous process of the 9th thoracic vertebra on the posterior mid - line.	The Du Meridian	
➅	Jizhong (DU6)	On the back, in the depression below the spinous process of the 11th thoracic vertebra on the posterior mid - line.	The Du Meridian	

**FIGURE 3:**
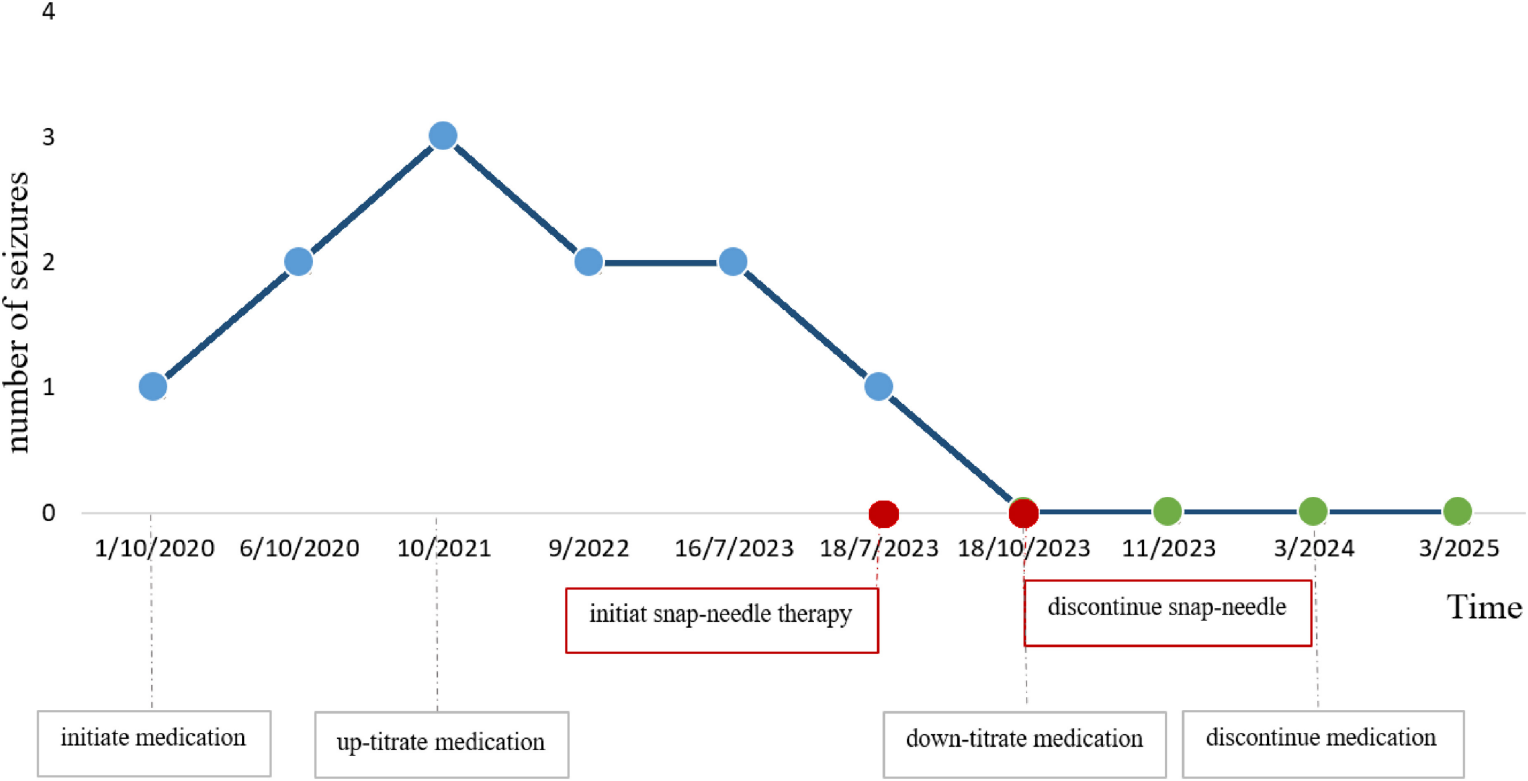
Line graph showing the number of seizures from October 2020 to March 2025. Seizures increase from 1 to 3 by October 2021, then decrease steadily to 0 by November 2023. Key events include initiating snap-needle therapy in July 2023 and discontinuing it in October 2023, resulting in seizure reduction. Medication adjustments are noted alongside.

Following treatment, the child’s condition stabilized, allowing for a gradual reduction in the dosage of levetiracetam tablets in collaboration with the neurology department. During follow-up until March 2025, the boy did not experience any further GTCSs. Meanwhile, parent interviews showed the child achieved a score of 98.4 on the Chinese version of the QOLCE-16-C quality of life scale (Tang et al., 2022) post-treatment. After 1 year of follow-up, parent reports showed the child’s daily function improved: nighttime sleep interruptions decreased from 3 to 0 per year; parents and teachers noted more stable emotions, independent homework completion (pre-intervention required full parental assistance), and increased peer interaction frequency from 1–2 to 5–6 times per day (per school teacher observation records). These findings indicate good recovery in the child (Figure 3 and Table 2).

**TABLE 2:** Structured abstract of epilepsy treatment process.

1. Initial onset and medication adjustment			
Time	Details of epileptic seizures	Treatment adjustment	Treatment response
October 1, 2020	A sudden GTCS occurred at night with no clear trigger, lasting about 2 min	Levetiracetam tablets were started, with a dosage of 0.375 g in the morning and 0.5 g in the evening	No records of post - treatment seizures
October 6, 2020	Two GTCSs occurred at night with no clear trigger, each lasting about 2–3 min	No adjustment of medication dosage	The frequency of s increased in the short term
A certain day in October 2021	Three GTCSs of epilepsy occurred with no clear trigger, each lasting about 3 min; an electroencephalogram (EEG) showed an increase in pathological waves	The dosage of levetiracetam tablets was adjusted to 0.75 g in the morning and 1 g in the evening	No records of post - treatment seizures
A certain day in September 2022	Two GTCSs of epilepsy occurred with no clear trigger, each lasting about 3 min	No adjustment of medication dosage	The frequency of seizures showed no significant improvement
**2. Pre - visit and acupuncture treatment**			
**Time**	**Details of epileptic seizures**	**Treatment adjustment**	**Treatment response**
July 16, 2023	Two GTCSs of epilepsy occurred with no clear trigger, each lasting about 3 min	No adjustment of the original drug treatment	No records of post - treatment seizures
July 18, 2023	No seizure	Three months of snap - needle treatment at acupoints was initiated	No records of post - treatment seizures
July 18, 2023 (first snap-needle treatment)	One GTCS of epilepsy occurred with no clear trigger, lasting about 2 min	The snap - needle treatment at acupoints was continued, and the medication dosage was not adjusted	The frequency of seizures was lower than before treatment
July 19, 2023–October 18, 2023	No seizure	The snap - needle treatment at acupoints was continued, and the medication dosage was not adjusted	No seizure
**3. Recovery and follow - up**			
**Time**	**Details of epileptic seizures**	**Treatment adjustment**	**Treatment response**
November 2023	No seizure	No adjustment of the treatment plan	The child’s condition recovered
March 2024	No seizure	Drug discontinuation	No recurrence of epilepsy
March 2025	As of now, no epileptic seizure	Drug discontinuation	No recurrence of epilepsy

## 3 Discussion

Benign epilepsy of childhood with central temporal spikes constitutes approximately 6%–7% of all childhood epilepsy cases (Wirrell et al., 2011), and its prognosis is generally regarded as favorable (Camfield and Camfield, 2012). Nevertheless, there is a potential risk of developing other, more refractory epilepsy syndromes (Taylor et al., 2008). BECTS exhibits familial aggregation, with relatives of individuals with BECTS showing a higher risk of epilepsy compared to control groups (Neubauer et al., 1998; De Tiège et al., 2006). Focal spikes associated with BECTS are typically activated during sleep (Camfield and Camfield, 2012), and sleep-related epileptic activity during sleep can disrupt physiological slow-wave activity (SWA). Local synapses undergo modification following sleep in the SWA steady state (Tononi and Cirelli, 2003) and performance on motor and categorization tasks improves after sleep (Fenn et al., 2003), such disruptions may impair neurons’ learning capacity (Tassinari and Rubboli, 2006). Conversely, BECTS may also cause cortical dysfunction, subsequently impairing cognitive function and hindering local plasticity changes in associated neurons (Aarts et al., 1984). Cortical regions impacted by epileptic activity in children with BECTS exhibit significant spiking, which predisposes these individuals to disturbances in frontal lobe function during childhood development. This disruption adversely affects the normal physiological processes underlying cognitive function. Consequently, children’s physical performance, intelligence quotient (IQ), memory, and executive function are notably compromised (Massa et al., 2001). At the molecular level, the presence of abundant spiking waves is attributed to the hyper-synchronized action of γ-aminobutyric acid (GABA), which inhibits the enhancement of cortical inhibition mediated by postsynaptic potentials. This heightened cortical inhibition temporarily disrupts the normal physiological processes essential for cognitive function (Massa et al., 2001), resulting in cognitive deficits in the affected child. Furthermore, previous research has indicated that a subset of BECTS patients experience neuropsychological and behavioral deficits of varying severity. The disorder also has long been associated with transient or permanent speech impairments (Scheffer et al., 1995).

In this particular case, the patient experienced initial symptom onset at the age of 7. The episodes were exclusively nocturnal, occurring during sleep (i.e., sleep-related). Prior to treatment initiation, a video EEG was performed, revealing abnormal waveforms characterized by short-range spikes, spikes and slow waves, polyspike-and-slow waves, with a pronounced presence on the right side. These abnormal discharges predominantly involved the central, middle temporal, and posterior temporal regions, with a marked increase during sleep. Brain magnetic resonance imaging (MRI) revealed no structural abnormalities. The child had no significant perinatal history, no history of febrile seizures, and no family history of epilepsy or epilepsy-related disorders. The seizure pattern showed a strong correlation with the sleep-wake cycle. Based on the combination of clinical history, symptomatology, examination findings, and family history, a diagnosis of BECTS syndrome was supported.

The patient exhibits a propensity for frequent short-term GTCSs, which have adversely impacted the child’s learning abilities and cognitive behavior, rendering them slightly below the normative levels for same-age children. These GTCSs have also significantly influenced the child’s self-confidence and psychosocial functioning. At the initial clinic visit, the child demonstrated marked low self-esteem and reluctance to interact with peers. Despite antiepileptic drug (AED) treatment, the child experienced five GTCSs prior to their initial consultation at the acupuncture department of our TCM hospital in 2023. During this period, the patient received multiple doses of levetiracetam. Suggesting that the disease remains uncontrolled and progressive. Numerous studies have demonstrated that the initial AED response and early treatment response, are strong prognostic indicators of favorable outcome (Sillanpää et al., 1998; Annegers et al., 1979; Elwes et al., 1984). This evidence suggests a higher likelihood of persistent abnormal EEG activity. Consequently, timely management of the patient’s seizure progression is of paramount importance. Failure to do so may result in the persistence of abnormal electroencephalographic activity, which could further impair the function of the corresponding cerebral cortex. This impairment may lead to abnormalities in the child’s motor skills, somatosensory processing, spatial awareness, and memory function.

In comparison to traditional acupuncture, snap needles are characterized by shorter lengths and more superficial insertion, resulting in gentler stimulation that is particularly suitable for pediatric patients. Additionally, snap-needle therapy enables prolonged treatment durations and minimizes disruption to daily activities, rendering it advantageous for conditions requiring long-term management. Basic research indicates that snap-needle application activates acupoint sensory neurons, which mediate signals to the central nervous system (Magnusson et al., 1994) and increase cortical gamma oscillations (Wu et al., 2024) -a process that may improve cognition, memory formation, and attention (Gillespie et al., 2016). However, this mechanism requires further validation via subsequent animal experiments or clinical studies. Recent neuroimaging studies has demonstrated that snap-needle stimulation of the vagus nerve distribution in the ear significantly activates the nucleus of the solitary tract (NTS) within the brainstem. This nerve impulse is subsequently transmitted through the reticular activating system to the limbic system (Frauscher et al., 2023). Current evidence suggests that snap needles establish a bioelectrical circuit via the auricular branch of the vagus nerve, and that 0.3–0.5 mA microcurrent stimulation can modulate autonomic homeostasis for up to 72 h (Percin et al., 2024). No molecular biology or electrophysiological tests (e.g., neurotransmitter level detection, intracerebral oscillatory recordings) were conducted to confirm the specific role of the aforementioned mechanism in this patient, requiring follow-up experimental verification.

Numerous mechanistic studies have demonstrated that acupoint stimulation can alleviate epilepsy by activating the vagus nerve (He et al., 2012; Cakmak, 2006), this activation subsequently stimulates the nucleus of the solitary tract (NTS) (Rhoton et al., 1966; Morest, 1967; Aghajanian and Wang, 1977; Ricardo and Koh, 1978; Saper and Loewy, 1980; Saper, 1982). This activation modulates opioid receptors in the amygdala, thereby suppressing focal epilepsy and preventing epilepsy-related sleep disturbances (Yi et al., 2015).

Regarding the selection of acupoints for epilepsy treatment, it is crucial individualize acupoint choice based on the patient’s specific condition, as different acupoints exert distinct effects on brain structural functions. This individualized approach aims to optimize therapeutic efficacy. In this case report, we used traditional acupoint localization combined with snap-needle therapy, targeting the patient’s back acupoints to reduce seizure frequency, improve the child’s learning and cognitive abilities, and potentially avoid the need for antiepileptic drug (AED) treatment. One of the primary points, DU14, is known to activate brain function and regulate cognitive function. Recent studies have shown that electroacupuncture (EA) stimulation at this point can effectively treat temporal lobe epilepsy (TLE) in rats. The underlying mechanism involves increased levels of p-ULK1/ULK1, LC3-II/LC3-I, and p62 in these rats following stimulation. Meanwhile, prior studies have shown that GV14 stimulation modulates the AKT/mTOR signaling pathway to treat epilepsy and promotes hippocampal neuron autophagy during epileptic seizures (Gao et al., 2022). The liver acupoint (BL18) primarily disperses liver wind, and previous studies have shown submerged needle stimulation of BL18 controls drug-resistant epileptic seizures (Nguyen et al., 2023). Additionally, stimulation of BL13 has been reported to enhance prefrontal learning and memory in rats, which is attributed to its role in downregulating the expression of IL-1β and TNF-α in the prefrontal cortex and hippocampus, thereby mitigating inflammatory responses (Wu et al., 2015). DU11 is frequently utilized to regulate nervous system function and demonstrates significant efficacy in treating mental disorders such as insomnia and anxiety. Earlier research indicates that stimulation of this acupoint can ameliorate sleep disorders by influencing neurotransmitter balance, specifically through the modulation of dopamine release in regions such as the nucleus ambiguous (Jin et al., 2018). DU14, DU11, DU8, and DU6 are situated along are located on the Du Meridian (adjacent to the spine). Studies have demonstrated that stimulating the Du Meridian activates adjacent spinal and sympathetic nerves, which inhibits cerebral cortical overexcitation, enhances the release of γ-aminobutyric acid (GABA, the central nervous system’s primary inhibitory neurotransmitter), and reduces abnormal neuronal discharge via nerve pathways (Linderoth et al., 1994).

## 4 Conclusion

In conclusion, our case study suggests that, in conjunction with medication, the use of snap needles can enhance the ability of children with epilepsy to live and learn, improve their quality of life, and, most importantly, reduce seizure frequency. Snap needles may represent an effective, cost-efficient, and safe adjunctive therapy. However, this report has notable limitations. First, despite the progressive reduction in oral levetiracetam dosage, the confounding effect of this medication on seizure remission cannot be fully excluded. Second, while snap-needle therapy may have shortened the child’s BECTS duration, spontaneous resolution–consistent with BECTS’ self-limiting nature–remains a possibility. Thus, this report only puts forward a hypothesis based on a single case: snap-needle intervention may be associated with BECTS seizure remission. Subsequent studies should eliminate drug confounders, include EEG findings as a core outcome measure, and adopt larger sample sizes to systematically evaluate the therapy’s effectiveness.

## Data Availability

The original contributions presented in this study are included in this article/supplementary material, further inquiries can be directed to the corresponding author.
